# Crystal structure of (*Z*)-7,8-di­chloro-4-(2-oxo­propyl­idene)-4,5-di­hydro-1*H*-1,5-benzodiazepin-2(3*H*)-one

**DOI:** 10.1107/S2056989015023750

**Published:** 2015-12-16

**Authors:** Sanae Lahmidi, Abdelhanine Essaghouani, El Mokhtar Essassi, Mohamed Saadi, Lahcen El Ammari

**Affiliations:** a Laboratoire de Chimie Organique Hétérocyclique URAC 21, Pôle de Compétence Pharmacochimie, Av. Ibn Battouta, BP 1014, Faculté des Sciences, Université Mohammed V, Rabat, Morocco; b Laboratoire de Chimie du Solide Appliquée, Faculté des Sciences, Université Mohammed V de Rabat, Avenue Ibn Battouta, BP 1014, Rabat, Morocco

**Keywords:** crystal structure, 2-oxo­propyl­idene, 1,5-benzodiazepinone, hydrogen bonding

## Abstract

In the title compound, C_12_H_10_Cl_2_N_2_O_2_, the seven-membered heterocycle displays a half-chair conformation. The mean plane through the oxo­propyl­idene group makes a dihedral angle of 36.44 (9)° with the fused benzene ring. An intra­molecular N—H⋯O hydrogen bond to close an *S*(6) loop is noted. An important feature of the mol­ecular packing are N—H⋯O hydrogen bonds that lead to the formation of helical supra­molecular chains along the *b* axis.

## Related literature   

For the pharmaceutical and biochemical properties of 1,5-benzodiazepine and their derivatives, see: El Azzaoui *et al.* (1999 ▸); Gringauz (1999 ▸); Swamy *et al.* (2008 ▸). For related structures, see: El Abbassi *et al.* (1997 ▸); Akkurt *et al.* (2006 ▸).
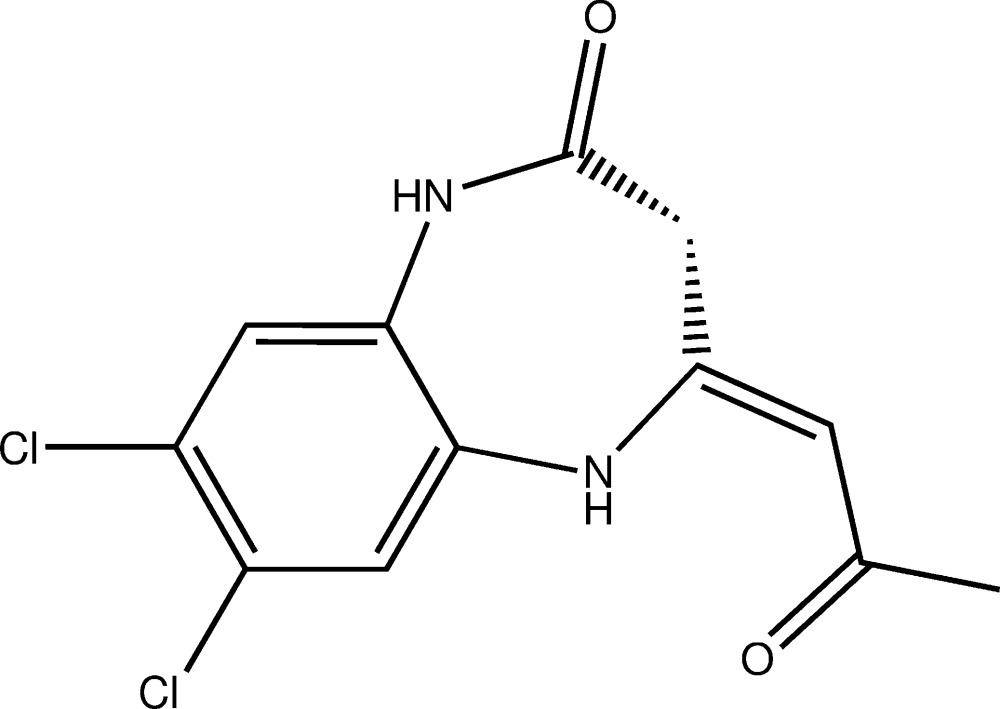


## Experimental   

### Crystal data  

C_12_H_10_Cl_2_N_2_O_2_
*M* *_r_* = 285.12
Monoclinic, 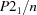

*a* = 7.6789 (4) Å
*b* = 13.2199 (6) Å
*c* = 12.4129 (7) Å
β = 102.561 (3)°
*V* = 1229.93 (11) Å^3^
*Z* = 4
Mo *K*α radiation
μ = 0.52 mm^−1^
*T* = 296 K
0.36 × 0.33 × 0.24 mm

### Data collection  

Bruker X8 APEX diffractometer
Absorption correction: multi-scan (*SADABS*; Bruker, 2009 ▸) *T*_min_ = 0.672, *T*_max_ = 0.746
25693 measured reflections
3299 independent reflections
2692 reflections with *I* > 2σ(*I*)
*R*_int_ = 0.031

### Refinement  

*R*[*F* ^2^ > 2σ(*F* ^2^)] = 0.038
*wR*(*F* ^2^) = 0.112
*S* = 1.02
3298 reflections
163 parameters
H-atom parameters constrained
Δρ_max_ = 0.36 e Å^−3^
Δρ_min_ = −0.33 e Å^−3^

### 

Data collection: *APEX2* (Bruker, 2009 ▸); cell refinement: *SAINT-Plus* (Bruker, 2009 ▸); data reduction: *SAINT-Plus*; program(s) used to solve structure: *SHELXS97* (Sheldrick, 2008 ▸); program(s) used to refine structure: *SHELXL2014* (Sheldrick, 2015 ▸); molecular graphics: *ORTEP-3 for Windows* (Farrugia, 2012 ▸); software used to prepare material for publication: *PLATON* (Spek, 2009 ▸) and *publCIF* (Westrip, 2010 ▸).

## Supplementary Material

Crystal structure: contains datablock(s) I. DOI: 10.1107/S2056989015023750/tk5415sup1.cif

Structure factors: contains datablock(s) I. DOI: 10.1107/S2056989015023750/tk5415Isup2.hkl

Supporting information file. DOI: 10.1107/S2056989015023750/tk5415Isup3.cml

**Fig. 1.**
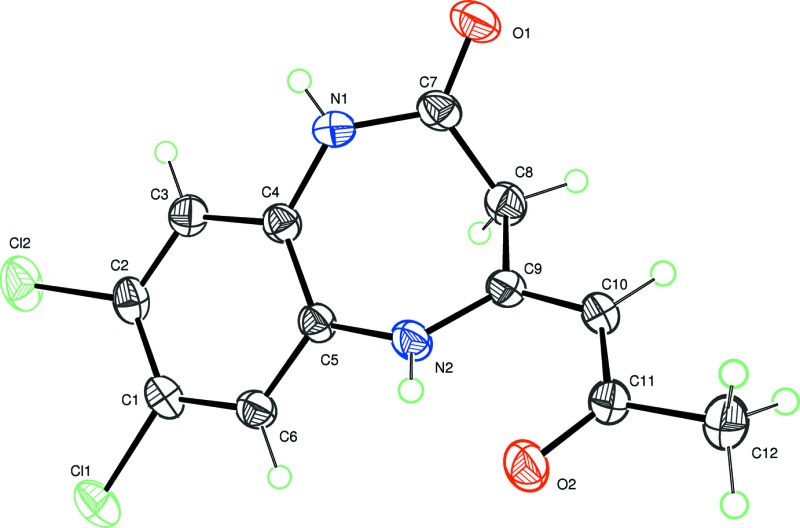
Molecular structure of the title compound with the atom-labelling scheme. Displacement ellipsoids are drawn at the 50% probability level. H atoms are represented as small circles.

. DOI: 10.1107/S2056989015023750/tk5415fig1.tif
Mol­ecular structure of the title compound with the atom-labelling scheme. Displacement ellipsoids are drawn at the 50% probability level. H atoms are represented as small circles.

**Fig. 2.**
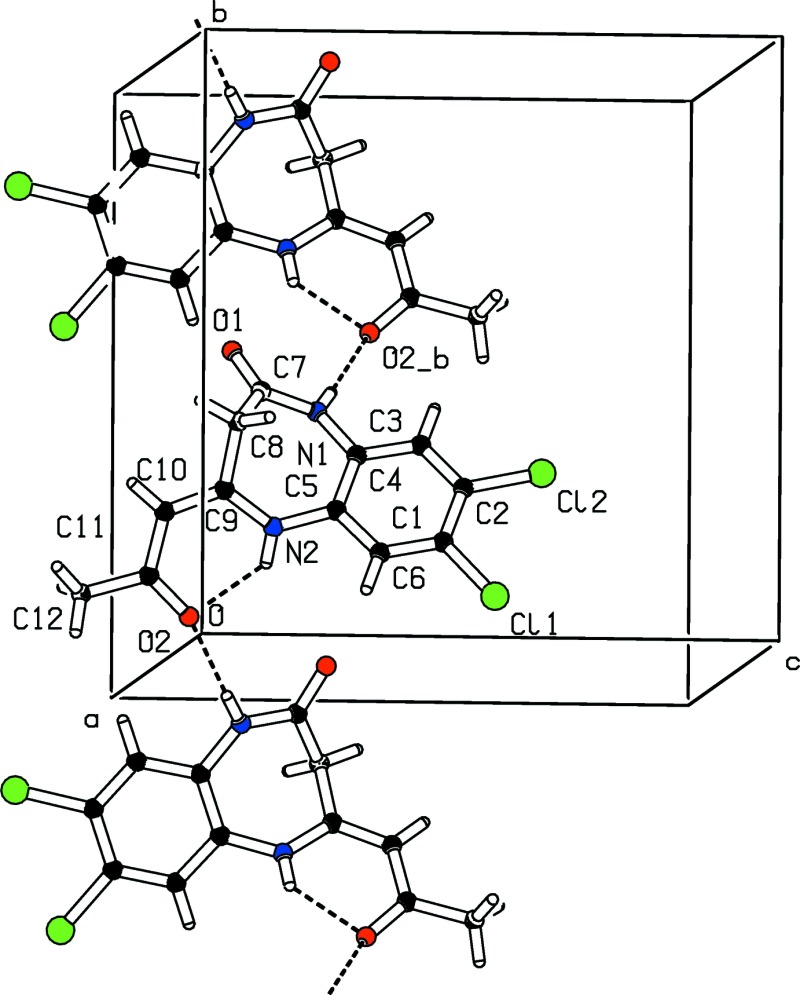
Structure of the title compound, showing molecules linked through N1—H1···O1 hydrogen bonds and the intramolecular hydrogen bond N2—H2···O2 (dashed lines).

. DOI: 10.1107/S2056989015023750/tk5415fig2.tif
Structure of the title compound, showing mol­ecules linked through N1—H1⋯O1 hydrogen bonds and the intra­molecular hydrogen bond N2—H2⋯O2 (dashed lines).

CCDC reference: 1441702

Additional supporting information:  crystallographic information; 3D view; checkCIF report

## Figures and Tables

**Table 1:** Hydrogen-bond geometry (Å, °)

*D*—H⋯*A*	*D*—H	H⋯*A*	*D*⋯*A*	*D*—H⋯*A*
N2—H2⋯O2	0.86	1.96	2.6410 (18)	135
N1—H1⋯O2^i^	0.86	1.95	2.8010 (19)	173

Symmetry code: (i) 
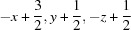
.

## supplementary crystallographic information

### S1. Comment

1,5-Benzodiazepines are used as starting materials in the synthesis of several heterocyclic compounds studied for potential biological activities (El Azzaoui *et al.* 1999). They are used for the purpose of hypnotic effects, owing to their less toxic and less severe withdrawal effects when compared with barbiturates (Gringauz, 1999). Some benzodiazepine derivatives have been widely used as anti-bacterial, anti-fungal, analgesic and anti-convulsant agents (Swamy *et al.*, 2008). In our laboratory we were interested in the synthesis of new 1,5-benzodiazepine derivatives (El Abbassi *et al.*, 1997; Akkurt *et al.*, 2006). The purpose of this work is to synthesize (*Z*)-7,8-dichloro-4,5-dihydro-4-(2-oxopropylidene)-1*H*-benzo[*b*][1,4] diazepin-2(3*H*)-one by condensation of 4,5-dichloro-*o*-phenylenediamine with 4-hydroxy-6-methyl-2*H*-pyran-2-one.

The molecule of the title compound, Fig. 1, is build up from two fused six- and seven-membered rings linked to a 2-oxopropylidene group. The seven-membered ring displays a half-chair conformation as indicated by the puckering amplitude QT = 0.811 (2) Å and spherical polar angle θ2 = 73.9 (2)°, φ2 = 129.07 (12)° and φ3 = −76.3 (4)°. Moreover, the dihedral angle between the mean plane through the oxopropylidene group and the dichlorobenzene ring is of 36.44 (9)°.

In the crystal, the molecules are linked by hydrogen bonds in the way to build an helical chain along the *b* axis as shown in Fig. 2 and Table 1. An intramolecular hydrogen bond N2—H2···O2 is also observed in this structure.

### S2. Experimental

A mixture of 4,5-dichloro-*o*-phenylenediamine (1.13 g) and of 4-hydroxy-6-methyl-2*H*- pyran-2-one (0.25 g) in xylene (30 ml) was heated at reflux for 4 h with azeotropic distillation. The completion of the reaction was confirmed by TLC. The solid obtained upon cooling the mixture was recrystallized from ethanol to afford colourless crystals in 75% yield.

### S3. Refinement

The H atoms were located in a difference map and treated as riding with C—H = 0.93–0.97 Å and N—H = 0.86 Å, and with *U*_iso_(H) = 1.2–1.5*U*_eq_(C, N).

### Crystal data

**Table:**

C_12_H_10_Cl_2_N_2_O_2_	*F*(000) = 584
*M**_r_* = 285.12	*D*_x_ = 1.540 Mg m^−^^3^
Monoclinic, *P*2_1_/*n*	Mo *K*α radiation, λ = 0.71073 Å
*a* = 7.6789 (4) Å	Cell parameters from 3299 reflections
*b* = 13.2199 (6) Å	θ = 2.3–29.1°
*c* = 12.4129 (7) Å	µ = 0.52 mm^−^^1^
β = 102.561 (3)°	*T* = 296 K
*V* = 1229.93 (11) Å^3^	Block, colourless
*Z* = 4	0.36 × 0.33 × 0.24 mm

### Data collection

**Table:**

Bruker X8 APEX diffractometer	3299 independent reflections
Radiation source: fine-focus sealed tube	2692 reflections with *I* > 2σ(*I*)
Graphite monochromator	*R*_int_ = 0.031
φ and ω scans	θ_max_ = 29.1°, θ_min_ = 2.3°
Absorption correction: multi-scan (*SADABS*; Bruker, 2009)	*h* = −10→10
*T*_min_ = 0.672, *T*_max_ = 0.746	*k* = −18→18
25693 measured reflections	*l* = −16→16

### Refinement

**Table:**

Refinement on *F*^2^	0 restraints
Least-squares matrix: full	Hydrogen site location: difference Fourier map
*R*[*F*^2^ > 2σ(*F*^2^)] = 0.038	H-atom parameters constrained
*wR*(*F*^2^) = 0.112	*w* = 1/[σ^2^(*F*_o_^2^) + (0.0553*P*)^2^ + 0.6111*P*] where *P* = (*F*_o_^2^ + 2*F*_c_^2^)/3
*S* = 1.02	(Δ/σ)_max_ = 0.001
3298 reflections	Δρ_max_ = 0.36 e Å^−^^3^
163 parameters	Δρ_min_ = −0.33 e Å^−^^3^

### Special details

**Table:**

Geometry. All e.s.d.'s (except the e.s.d. in the dihedral angle between two l.s. planes) are estimated using the full covariance matrix. The cell e.s.d.'s are taken into account individually in the estimation of e.s.d.'s in distances, angles and torsion angles; correlations between e.s.d.'s in cell parameters are only used when they are defined by crystal symmetry. An approximate (isotropic) treatment of cell e.s.d.'s is used for estimating e.s.d.'s involving l.s. planes.

### Fractional atomic coordinates and isotropic or equivalent isotropic displacement parameters (Å^2^)

**Table:**

	*x*	*y*	*z*	*U*_iso_*/*U*_eq_	
C1	0.5232 (2)	0.21313 (13)	0.49879 (13)	0.0338 (3)	
C2	0.5740 (2)	0.30850 (13)	0.54157 (13)	0.0355 (3)	
C3	0.6157 (2)	0.38307 (12)	0.47350 (13)	0.0346 (3)	
H3	0.6479	0.4470	0.5024	0.042*	
C4	0.6104 (2)	0.36438 (11)	0.36221 (13)	0.0303 (3)	
C5	0.5633 (2)	0.26785 (11)	0.31938 (12)	0.0291 (3)	
C6	0.5189 (2)	0.19345 (12)	0.38896 (13)	0.0330 (3)	
H6	0.4858	0.1294	0.3607	0.040*	
C7	0.6022 (2)	0.46740 (11)	0.19419 (14)	0.0349 (3)	
C8	0.4552 (2)	0.40089 (12)	0.13165 (15)	0.0375 (4)	
H8A	0.3586	0.3984	0.1707	0.045*	
H8B	0.4087	0.4298	0.0593	0.045*	
C9	0.5204 (2)	0.29534 (11)	0.11872 (13)	0.0310 (3)	
C10	0.5298 (2)	0.25845 (12)	0.01690 (13)	0.0333 (3)	
H10	0.4958	0.3007	−0.0440	0.040*	
C11	0.5889 (2)	0.15901 (12)	−0.00056 (13)	0.0330 (3)	
C12	0.6037 (3)	0.12898 (15)	−0.11491 (14)	0.0424 (4)	
H12A	0.5680	0.1847	−0.1645	0.064*	
H12B	0.7249	0.1111	−0.1145	0.064*	
H12C	0.5275	0.0720	−0.1388	0.064*	
N1	0.6678 (2)	0.44232 (10)	0.30134 (12)	0.0352 (3)	
H1	0.7364	0.4859	0.3410	0.042*	
N2	0.5688 (2)	0.23921 (10)	0.21168 (11)	0.0339 (3)	
H2	0.6012	0.1781	0.2028	0.041*	
Cl1	0.46370 (7)	0.11838 (4)	0.57991 (4)	0.04762 (15)	
Cl2	0.58703 (8)	0.33611 (4)	0.67921 (4)	0.05696 (17)	
O1	0.6604 (2)	0.54018 (9)	0.15280 (11)	0.0496 (3)	
O2	0.6297 (2)	0.09619 (9)	0.07595 (10)	0.0451 (3)	

### Atomic displacement parameters (Å^2^)

**Table:**

	*U*^11^	*U*^22^	*U*^33^	*U*^12^	*U*^13^	*U*^23^
C1	0.0353 (8)	0.0360 (8)	0.0318 (7)	0.0068 (6)	0.0109 (6)	0.0083 (6)
C2	0.0383 (9)	0.0408 (8)	0.0278 (7)	0.0116 (7)	0.0080 (6)	0.0008 (6)
C3	0.0383 (9)	0.0313 (7)	0.0330 (8)	0.0052 (6)	0.0053 (6)	−0.0031 (6)
C4	0.0316 (8)	0.0283 (7)	0.0309 (7)	0.0032 (6)	0.0066 (6)	0.0017 (6)
C5	0.0317 (8)	0.0281 (7)	0.0278 (7)	0.0031 (6)	0.0069 (6)	0.0018 (5)
C6	0.0389 (9)	0.0276 (7)	0.0330 (8)	0.0009 (6)	0.0089 (6)	0.0022 (6)
C7	0.0438 (9)	0.0245 (7)	0.0383 (8)	0.0055 (6)	0.0136 (7)	0.0015 (6)
C8	0.0410 (9)	0.0325 (8)	0.0370 (8)	0.0075 (7)	0.0043 (7)	0.0044 (6)
C9	0.0328 (8)	0.0278 (7)	0.0309 (7)	−0.0020 (6)	0.0039 (6)	0.0031 (6)
C10	0.0407 (9)	0.0315 (7)	0.0258 (7)	−0.0023 (6)	0.0029 (6)	0.0055 (6)
C11	0.0353 (8)	0.0346 (8)	0.0273 (7)	−0.0037 (6)	0.0028 (6)	0.0009 (6)
C12	0.0495 (10)	0.0468 (10)	0.0306 (8)	−0.0034 (8)	0.0083 (7)	−0.0031 (7)
N1	0.0416 (8)	0.0275 (6)	0.0359 (7)	−0.0056 (5)	0.0073 (6)	−0.0001 (5)
N2	0.0487 (8)	0.0253 (6)	0.0287 (6)	0.0033 (5)	0.0104 (6)	0.0020 (5)
Cl1	0.0591 (3)	0.0464 (3)	0.0420 (2)	0.0054 (2)	0.0213 (2)	0.01498 (18)
Cl2	0.0855 (4)	0.0567 (3)	0.0300 (2)	0.0141 (3)	0.0155 (2)	−0.00200 (18)
O1	0.0731 (10)	0.0323 (6)	0.0465 (7)	−0.0059 (6)	0.0199 (7)	0.0064 (5)
O2	0.0680 (9)	0.0334 (6)	0.0318 (6)	0.0095 (6)	0.0061 (6)	0.0038 (5)

### Geometric parameters (Å, º)

**Table:**

C1—C6	1.381 (2)	C8—C9	1.503 (2)
C1—C2	1.391 (2)	C8—H8A	0.9700
C1—Cl1	1.7297 (16)	C8—H8B	0.9700
C2—C3	1.380 (2)	C9—N2	1.3541 (19)
C2—Cl2	1.7286 (17)	C9—C10	1.371 (2)
C3—C4	1.395 (2)	C10—C11	1.422 (2)
C3—H3	0.9300	C10—H10	0.9300
C4—C5	1.399 (2)	C11—O2	1.2491 (19)
C4—N1	1.404 (2)	C11—C12	1.502 (2)
C5—N2	1.3989 (19)	C12—H12A	0.9600
C5—C6	1.399 (2)	C12—H12B	0.9600
C6—H6	0.9300	C12—H12C	0.9600
C7—O1	1.220 (2)	N1—H1	0.8599
C7—N1	1.357 (2)	N2—H2	0.8600
C7—C8	1.506 (2)		
			
C6—C1—C2	119.49 (15)	C9—C8—H8B	109.3
C6—C1—Cl1	119.04 (13)	C7—C8—H8B	109.3
C2—C1—Cl1	121.46 (13)	H8A—C8—H8B	108.0
C3—C2—C1	119.79 (15)	N2—C9—C10	122.05 (14)
C3—C2—Cl2	118.88 (13)	N2—C9—C8	116.99 (14)
C1—C2—Cl2	121.34 (13)	C10—C9—C8	120.95 (14)
C2—C3—C4	121.26 (15)	C9—C10—C11	123.48 (14)
C2—C3—H3	119.4	C9—C10—H10	118.3
C4—C3—H3	119.4	C11—C10—H10	118.3
C3—C4—C5	119.15 (14)	O2—C11—C10	122.29 (15)
C3—C4—N1	117.25 (14)	O2—C11—C12	119.00 (15)
C5—C4—N1	123.37 (14)	C10—C11—C12	118.71 (15)
N2—C5—C4	123.42 (14)	C11—C12—H12A	109.5
N2—C5—C6	117.50 (14)	C11—C12—H12B	109.5
C4—C5—C6	118.95 (14)	H12A—C12—H12B	109.5
C1—C6—C5	121.34 (15)	C11—C12—H12C	109.5
C1—C6—H6	119.3	H12A—C12—H12C	109.5
C5—C6—H6	119.3	H12B—C12—H12C	109.5
O1—C7—N1	120.91 (17)	C7—N1—C4	127.87 (14)
O1—C7—C8	123.03 (16)	C7—N1—H1	116.6
N1—C7—C8	116.06 (14)	C4—N1—H1	114.1
C9—C8—C7	111.53 (14)	C9—N2—C5	127.32 (13)
C9—C8—H8A	109.3	C9—N2—H2	116.1
C7—C8—H8A	109.3	C5—N2—H2	116.5

### Hydrogen-bond geometry (Å, º)

**Table:**

*D*—H···*A*	*D*—H	H···*A*	*D*···*A*	*D*—H···*A*
N2—H2···O2	0.86	1.96	2.6410 (18)	135
N1—H1···O2^i^	0.86	1.95	2.8010 (19)	173

Symmetry code: (i) −*x*+3/2, *y*+1/2, −*z*+1/2.
