# Supplementary figures and images for: Crystal structure of (Z)-7,8-di­chloro-4-(2-oxo­propyl­idene)-4,5-di­hydro-1H-1,5-benzodiazepin-2(3H)-one
Source: Acta Crystallogr E Crystallogr Commun. 2015 Dec 16;71(Pt 12):o1059–60. doi: 10.1107/S2056989015023750 (PMC4719978; doi:10.1107/S2056989015023750)

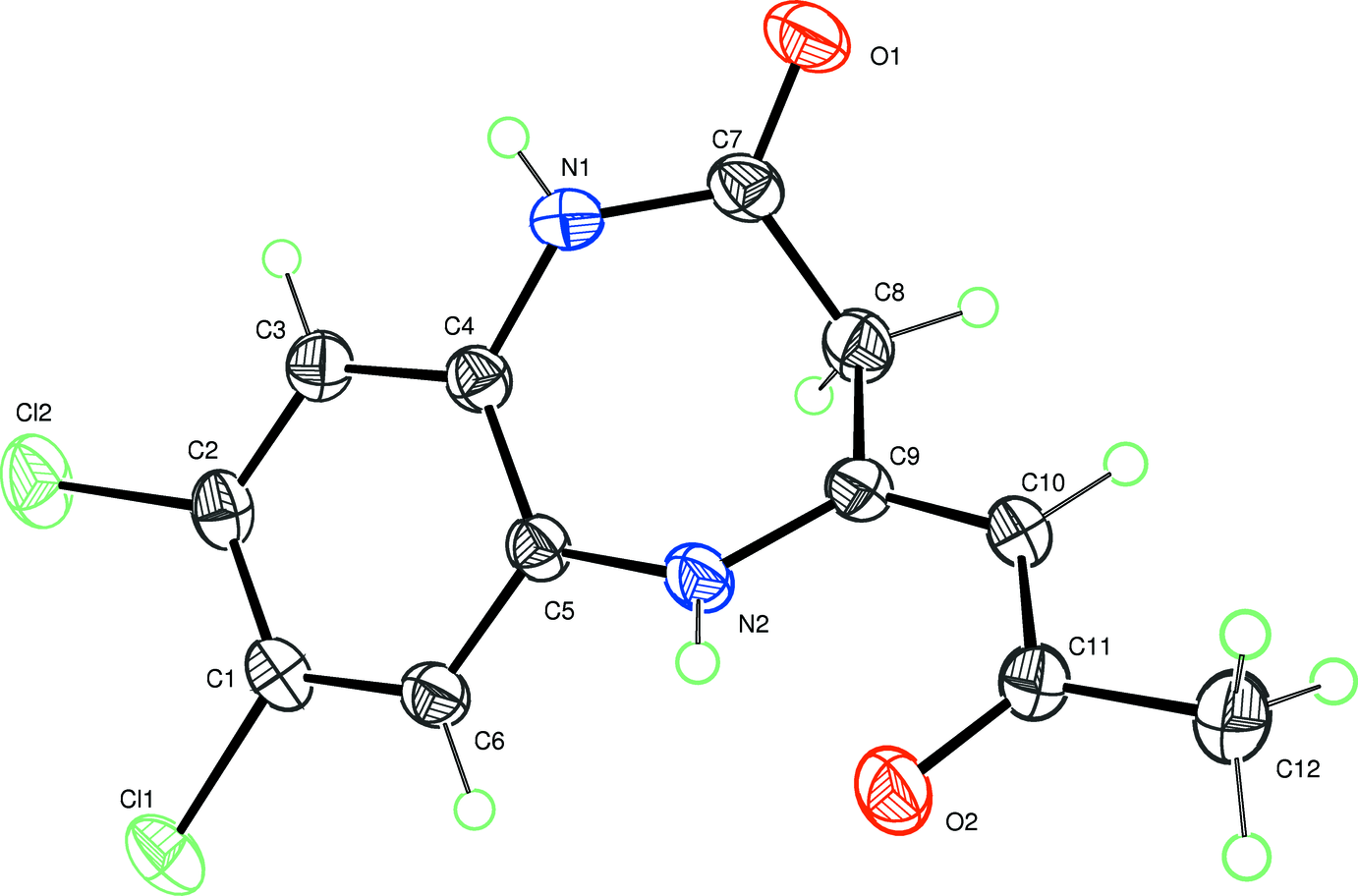

Supplement: Supplementary file 4 [file e-71-o1059-fig1.tif]

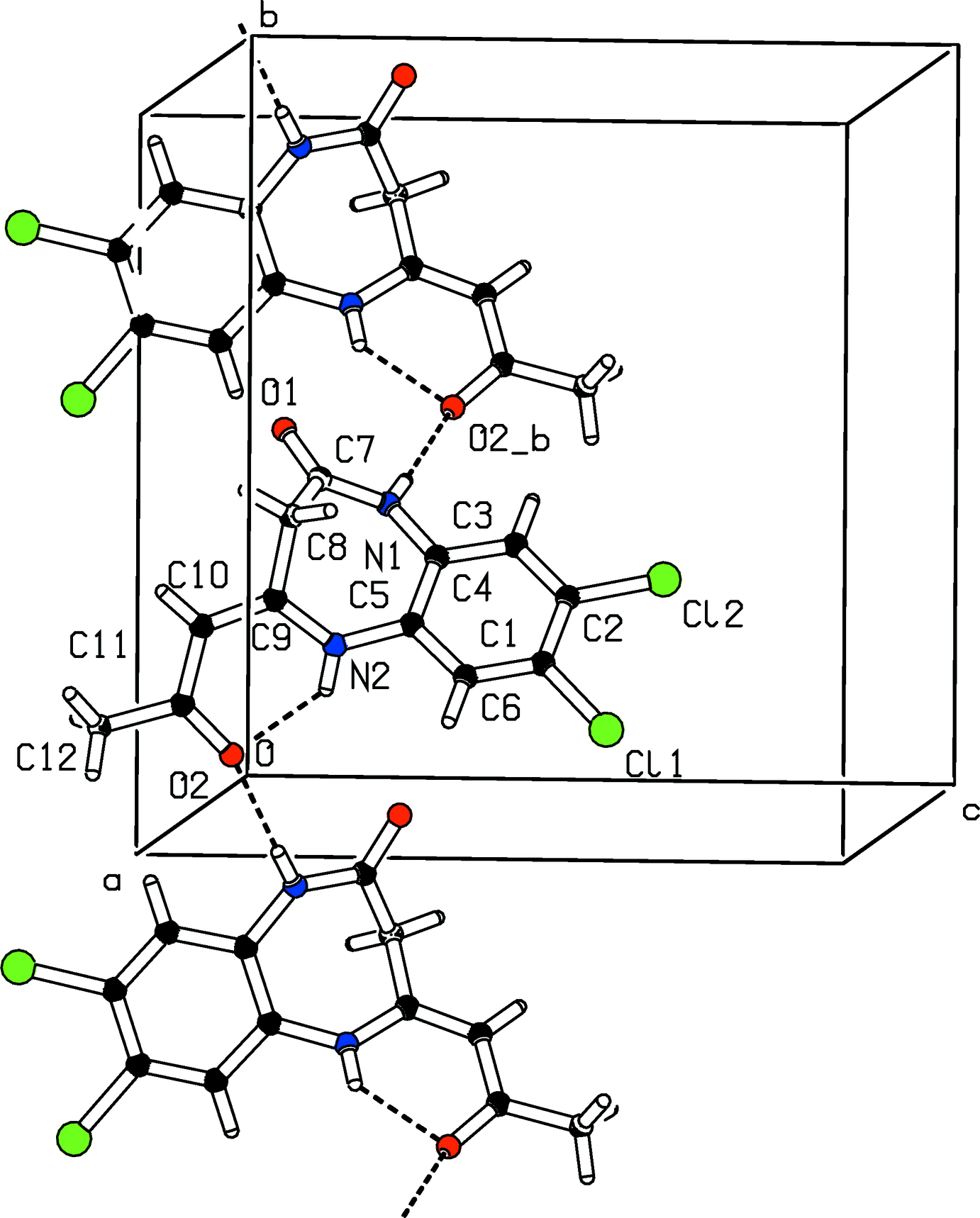

Supplement: Supplementary file 5 [file e-71-o1059-fig2.tif]
